# Experimental and mechanistic research on modifying the mechanic properties of the high water backfill material by electrochemical treatment

**DOI:** 10.1038/s41598-020-74115-8

**Published:** 2020-10-12

**Authors:** Shengrong Xie, Yaohui Sun, En Wang, Dongdong Chen, Xiaoyu Wu, Pengyu Qi

**Affiliations:** School of Energy and Mining Engineering, China University of Mining and Technology-Beijing, Beijing, 100083 China

**Keywords:** Mineralogy, Physical chemistry, Engineering, Civil engineering

## Abstract

To promote the engineering applications of high water backfill materials (HWBM) in mining, a series of experiments are performed to investigate the effects of the direct current (DC) electric field on the mechanic properties and electrical resistivity of HWBMs. Based on X-ray diffraction (XRD) and scanning electron microscopy (SEM) investigations, the influence of electrochemical treatment on the hydration products and the microstructure of the HWBM was studied. The results show that the peak strength, elastic modulus, deformation modulus and electrical resistivity of the HWBM samples all first increased and then decreased with the increasing of the potential gradient, and the peak points appeared when the potential gradient was 0.2 V/cm. The anisotropy of content of ettringite and calcium silicate hydrates (C–S–H) increased betweent the anodic and cathodic regions of samples. Meanwhile, microstructure in the anodic region of the samples was more stable after electrochemical treatment, which indicates that the different variation of mineralogical compositions and microstructures in different regions of the samples are the primary factors affecting the mechanic properties and electrical resistivity of the HWBM. Therefore, the electrochemical method is a potential technology to modify the engineering properties of the HWBM.

## Introduction

As an excellent mine backfilling material, the high water backfill material (HWBM) has the advantages of good liquidity, short initial setting time, and good early strength. However, the filling cost and strength of HWBM will increase with a decreasing the water cement ratio (W/C), while the strength will decrease with an increasing the W/C^1^. In addition, the disadvantage of poor toughness and small compressibility also limits the application of HWBMs for use as mine backfilling materials^2,3^.

To improve the performances of HWBMs and reduce the backfill cost, numerous studies have been performed, and the effects of using various additives (such as fly ash, gravel, river sludge, lithium carbonate, and aluminum sulfate) on the physical and chemical properties of HWBMs has been investigated^4–8^. The research shows that the uniaxial compression strength (UCS) and elastic modulus of the HWBM decreased after adding the fly ash, while the cost was reduced and the residual strength increased^4,5^. Additionally, the addition of lithium carbonate and aluminum sulfate can promote the hydration process of the HWBM^7,8^. However, electrochemical treatment techniques are also a new modified method applied to rock and soil mass, and can effectively improve the physicochemical properties of rock and soil mass. However, there are few reports on the electrochemical modification of backfill materials.

The electrochemical treatment technique is an interdisciplinary method, in which a direct electric current is applied via electrodes and passes through the rock or soil mass to modify their physical and chemical properties. The mechanisms of the electrochemical modification of rock and soil mass mainly consisted of electroosmosis, electro migration, and electrophoresis. By applying a direct electric field, the charged species in soil or rock mass (cations and anions, polar water and other charged particles) migrate towards the oppositely charged electrode, which significantly alters many of the physicochemical properties of the soil or rock mass^9–13^.

In recent years, the electrochemical method has been successfully applied to many fields of geotechnical engineering, such as the electrochemical consolidation of weak rock^10–12^, the electrochemical desalination of rock or soil^14–20^, the drainage and consolidation of soil by electro-osmosis^21–27^, and the acceleration of methane desorption in lump anthracite by the electrochemical treatment^28–30^. Meanwhile, the electrochemical treatment was used to accelerate the hydration reaction rate to enhance the strength and deformation characteristics of fly-ash-cemented filling materials^31^. These studies have proved that the electrochemical treatment is an effective technique for the modification of the physicochemical properties of rock and soil mass. However, there are no reports on the electrochemical modification of HWBM. To fill the research gaps, in this paper, we will focus on investigating the influence of electrochemical treatment on the strength and electrical resistivity of HWBMs.

Therefore, the objective of the present study was to evaluate the effects of the DC electric field on the mechanic properties and electrical resistivity of HWBMs, and the influence of the potential gradient on the modification efficiency of HWBMs was investigated. Meanwhile, XRD and SEM were used to investigate the influence of the electric field on the mineralogical composition and microstructure of different regions of the samples and to analyze the mechanism of electrochemical modification of the HWBM. Furthermore, the relationship between UCS and electrical resistivity of the HWBM was proposed.

## Materials and methods

### Materials

**Figure 1:**
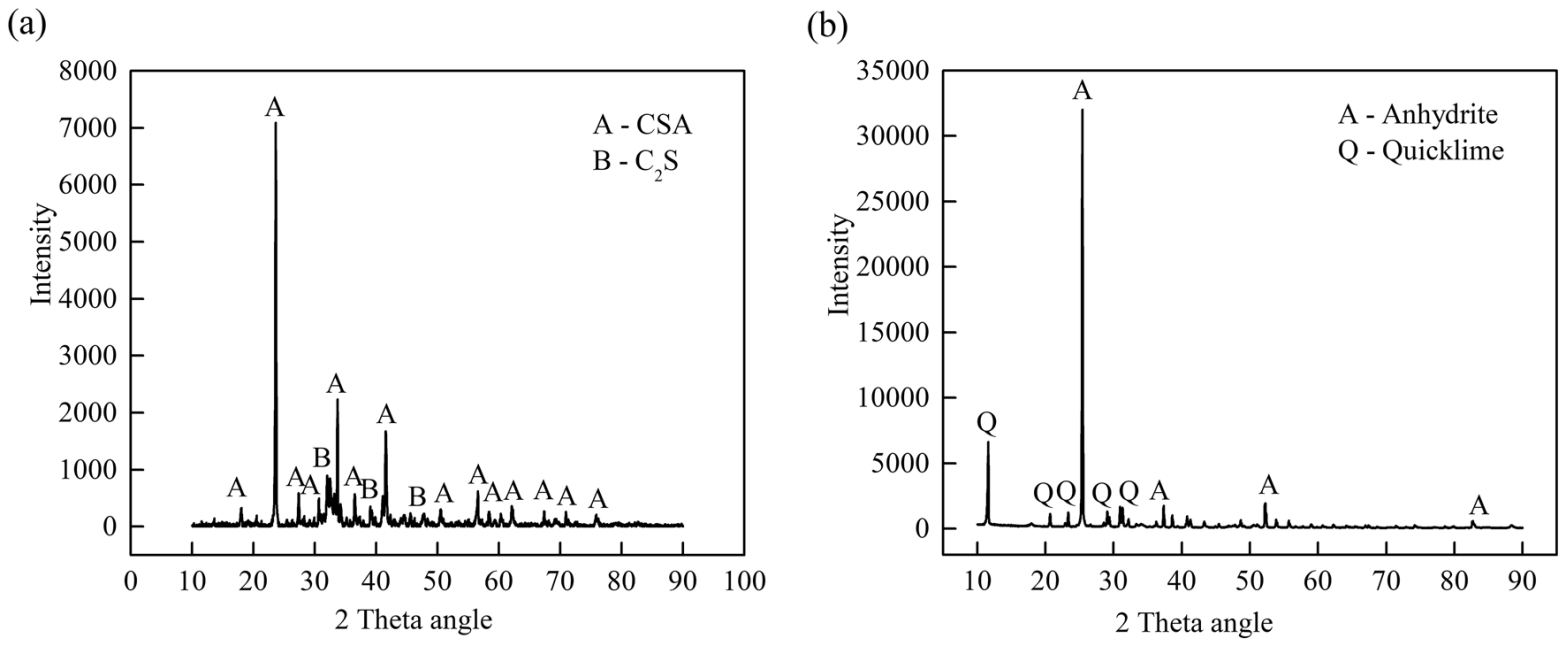
The XRD patterns of the binder materials of the HWBMs: (**a**) material A; (**b**) material B.

In this work, the HWBM was obtained from Hebei, China. The HWBM consisted of material A and material B. Figure 1 shows the XRD patternof materials A and BIt can been found that the main mineralogical composition in material A are calcium sulfoaluminate (CSA) of 68.15% and dicalcium silicate (C2S) of 31.85%, while the main minerals in material B are anhydrite of 74.85% and quicklime of 25.15%. The chemical composition of material A and B is shown in Tables 1 and 2. Meanwhile, compound additives were used as mineral additives, which included retarder, thickener and quicksetting agent, etc. Slurry A and B were prepared by adding water to material A and B, respectively with a ratio of 4:1, then the slurry A and B were mixed and stirred with a ratio of 1:1, finally formed HWBM consolidated body.

**Table 1:** Chemical compositon of material A (wt%).

Composition	CaO	Al_2_O_3_	SO_3_	SiO_2_	Fe_2_O_3_	MgO	Loss
Mass (%)	46.21	32.70	9.12	8.28	2.23	0.86	0.60

**Table 2:** Chemical compositon of anhydrite (wt%).

Composition	SO_3_	CaO	Al_2_O_3_	SiO_2_	Fe_2_O_3_	MgO	Loss
Mass (%)	54.22	34.38	1.46	3.06	1.36	3.02	2.50

### Experiment apparatus

**Figure 2:**
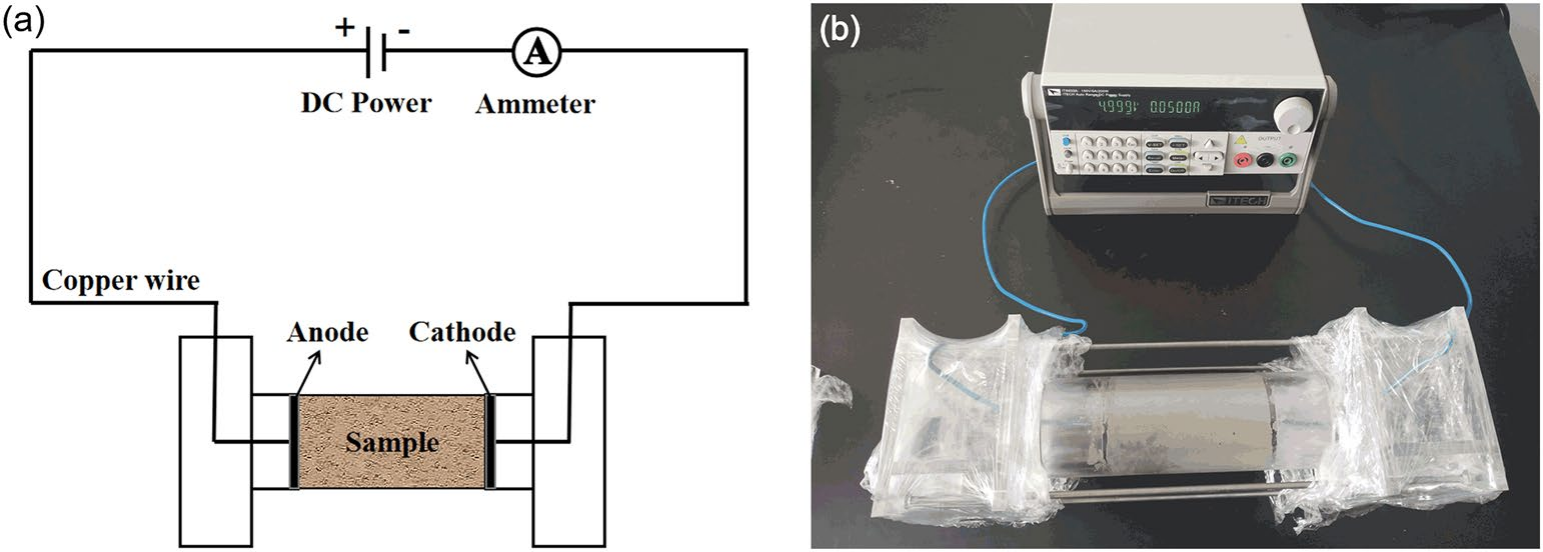
The electrochemical treatment applied to the HWBM. (**a**) Schematic diagram; (**b**) Photograph of the experiment system.

Three sets of electrochemical treatment experimental apparatuses were used, and each set consists primarily of a direct current (DC) power supply, an ammeter, an electrolytic cell, electrodes, copper wires and preliminary solidified HWBM samples. A schematic of electrochemical treatment applied to a HWBM is shown in Fig. 2a. The maximum output voltage and the maximum output current of the DC power supply (IT6933A, ITECH electronic Co Ltd, Nanjing, China) were 150 V and 5 A respectively. The electrode was a round porous iron plate with a diameter of 50 mm and thickness of 5 mm. The electrolytic cell was an acrylic tube with an internal diameter of 50 mm and a wall thickness of 10 mm, and both sides of the electrolytic cell consisted of an acrylic tube with an outer diameter of 50 mm and a rectangular acrylic box. Then, four threaded rods were used to connect and fix the electrolytic cell.

## Methods

### Preparation of samples

In this work, the proportion of material A to material B was 1:1, the ratio of water to material A and material B (the W/C of HWBMs) was 4:1 and the curing age was 7 days. Before the samples were prepared, a cylindrical mold with an inner diameter of 50 mm and a length of 200 mm was made to prepare the HWBM samples with diameters of 50 mm and lengths of 100 mm.

Materials A and B were mixed and stired with water to achieve a ratio of 4:1. Then, slurry A and slurry B were placed into molds and stirred. The HWBM samples were demolded after 2 h, then a plastic film was used to cover the samples to prevent weathering. Next, the samples were placed in a constant temperature and humidity room with a curing temperature of 20 °C and a humidity of 95%. Then, the samples were cured for 7 days. In this experiment, 18 samples were divided into six groups, including one control group and five testing groups.

### Electrochemical treatment

The HWBM samples were prepared as previously described. Then, the samples were placed into the acrylic tube of the electrolytic cell and electrode plates were placed on both ends of the sample. Next, each part of the electrochemical treatment apparatus was assembled as shown in Fig. 2b. The plastic film was used to seal the electrolytic cell to minimize the contact area of the sample with air.

After the electrochemical treatment apparatuses were assembled, the output power was regulated by the potential gradient, the output button of the DC power supply was pressed, and the timer was started. In this experiment, the power-on time was 3 h. Additionally, the potential gradient, a variable examined in this study, was 0.2 V/cm, 0.5 V/cm, 1.0 V/cm, 2.0 V/cm and 3.0 V/cm. After the electrochemical treatment, the samples were cured in the constant temperature and humidity room for 7 days.

### Electrical resistivity measurements

After curing of 7 days, the electrical resistivity of the HWBM samples was measured by a MCH2816A digital AC bridge (Shenzhen Meichuang Instrument Co. Ltd, China). Before the measurement was made, the two ends of the samples were smoothed using a face grinding machine, and the diameter and length of the samples were measured. Then, the resistance of the samples was measured at measurement frequencies of 50 Hz, 100 Hz, 500 Hz, 1 kHz, 5 kHz, 10 kHz, 50 kHz, 100 kHz and 200 kHz.

The electrical resistivity of a HWBM sample is calculated from Eq. (1)

1 $$ \rho = R \cdot S \cdot L^{ - 1} $$

where *R* is the electric resistence of the samples in *Ω*, *S* is the cross-sectional area of the samples in *m*^2^, and *L* is the length of the samples in *m*.

### Uniaxial compression tests

The UCS of the HWBM samples was measured using a WAW-600B electrohydraulic servo universal testing machine (Tianshui Hongshan Testing Machine Co, Ltd, China). According to the ISRM suggested methods, a loading rate of 2 mm/min was continually applied by controlling the axial strain until the specimen failed. Additionally, the elastic modulus and deformation modulus of the samples was calculated from the stress–strain curve.

### XRD and SEM tests

The XRD patterns of the samples were collected using an Aeris X-ray powder diffractometer (PANalytical Corporation). MDI Jade 6.0 software was used to obtain the mineralogical composition and perform the semiquantitative analysis on the anodic and cathodic regions of the HWBM samples. SEM was used to observe the effects of the electrochemical treatment on the surface morphologies at the anodic and cathodic regions of the HWBM samples.

## Results

### Change in the electric current on the electrochemical treatment process

**Figure 3:**
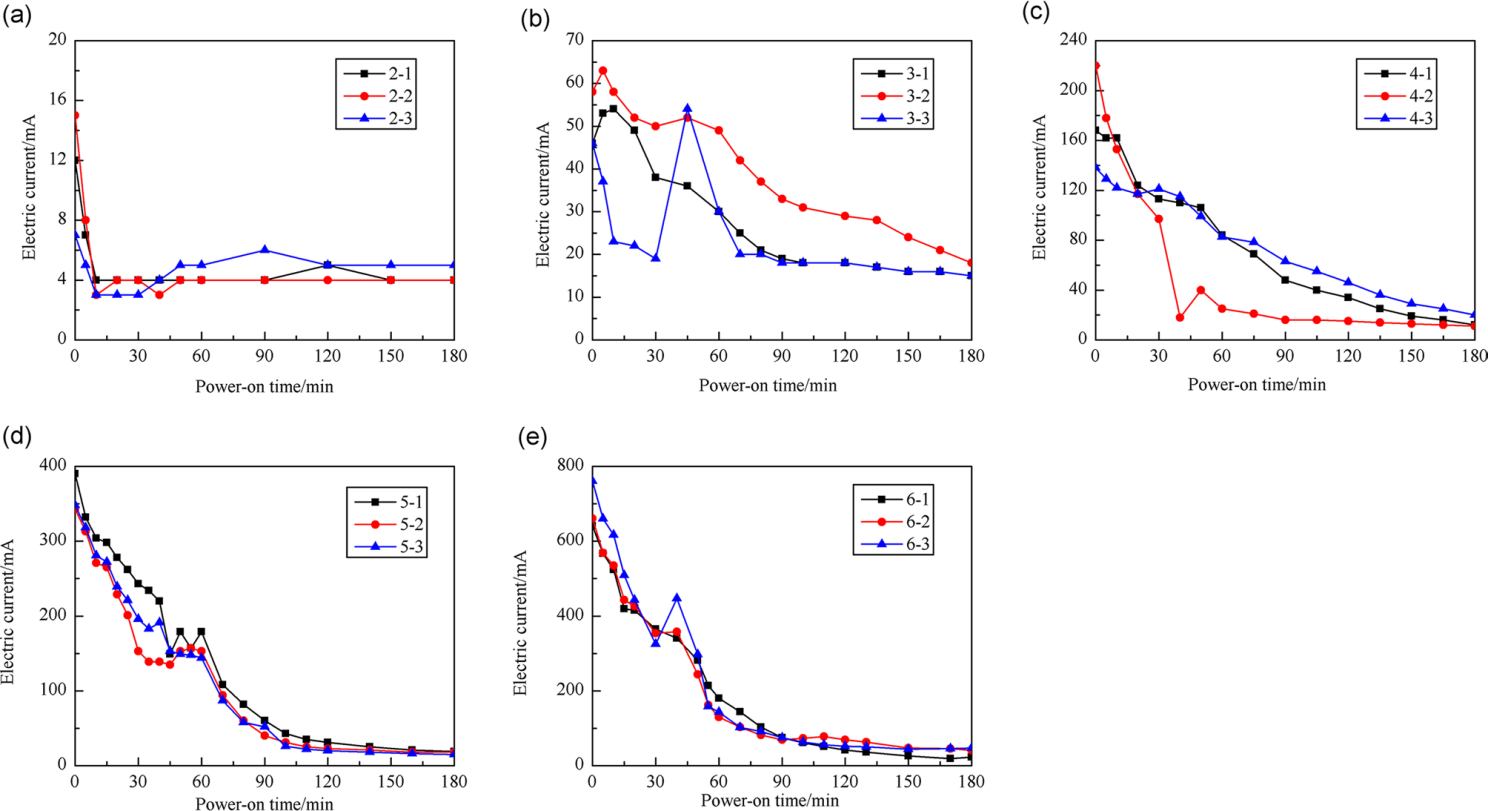
The electric current change through the samples during the electrochemical treatment process. (**a**) 0.2 V/cm; (**b**) 0.5 V/cm; (**c**) 1.0 V/cm; (**d**) 2.0 V/cm; (**e**)3.0 V/cm.

The change in the electric current through the HWBM samples measured during the electrochemical treatment is shown in Fig. 3.

**Figure 4:**
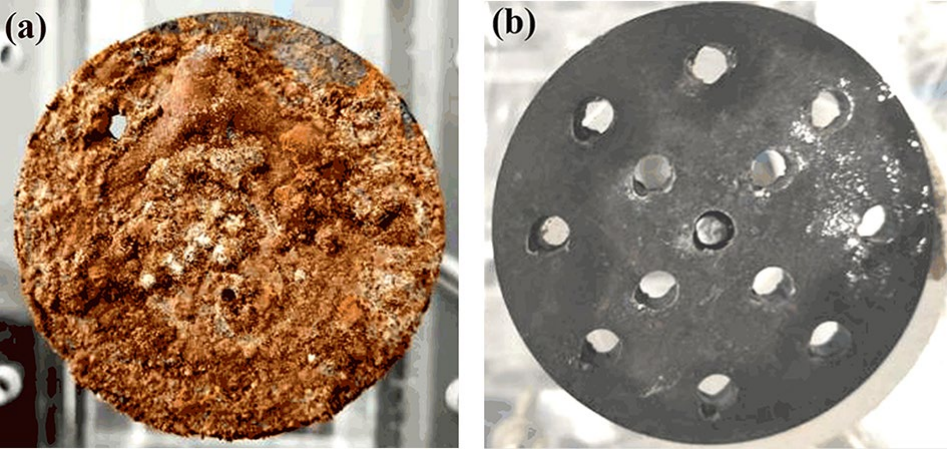
Electrode change after the electrochemical experiments: (**a**) the surface ot anode; (**b**) the surface of cathode.

As shown in Fig. 3a–e, during the electrochemical treatment process, the electric current through the HWBM samples had a tendency to rapidly decline at the early stage of the process, then slowly decline, and finally maintain a steady state. This phenomenon is explained by the hydration of the HWBM: during the early stage of the electrochemical treatment, the free ions and polar water molecules inside the sample directionally migrated along the electric field. Then, the number of ions and water molecules decreased as the HWBM was hydrated. Additionally, the rate at which the electric current decreased also decreased with increasing hydration product content. In the end, the basically stable electric current indicates that the HWBM was close to being completely hydrated. Additionally, the initial electric current increased due to the increasing of the potential gradient. Then the electric current rapidly declined and tended to stable when the potential gradient was 0.2 V/cm as shown in Fig. 4a, it may because that the internal free ions and polar water molecules of samples rapidly decreased with rapid initial setting of the HWBM, thus leading to slow charge migration under slow-valtage.

### Change in the surface of electrodes

**Figure 5:**
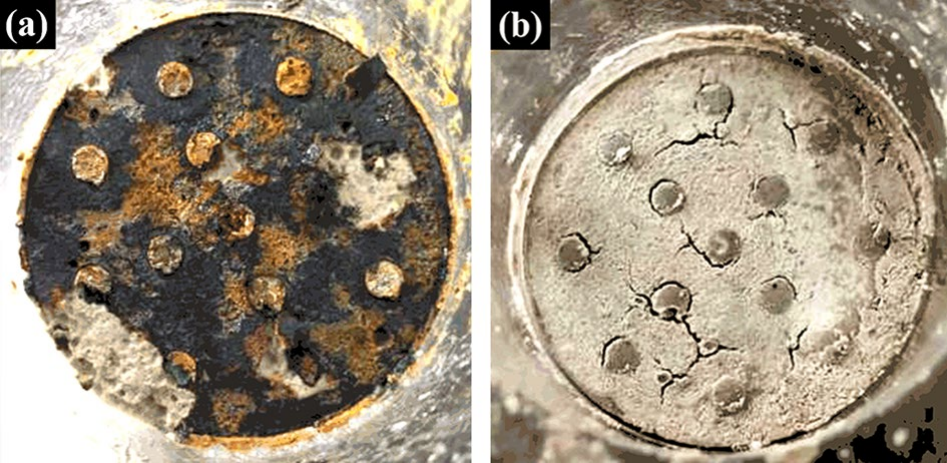
Change of the sample surfaces after the electrochemical treatment; (**a**) surface of anodic region; (**b**) surface of cathodic region.

Figure 4 shows that the surface of anodes were corroded after the electrochemical treatment, while the surface of cathodes were not obviously corroded. This difference is due to the oxidation reaction occurring in the anode and the reduction reaction occurring in the cathode under an applied electric field. Further oxidation of the anodic oxidation products formed a red brown iron oxide, as shown in Fig. 5a.

Additionally, the reduction of the water at the cathode produced hydrogen.

The reaction equations are as follows:

- Anodic:
  2 $$ {\text{Fe}} - 2{\text{e}}^{ - 1} \to {\text{Fe}}^{2 + } $$
  3 $$ {\text{Fe}}^{{{2} + }} + {\text{2OH}}^{ - } \to {\text{Fe}}\left( {{\text{OH}}} \right)_{{2}} \downarrow $$
  4 $$ {\text{4Fe}}\left( {{\text{OH}}} \right)_{{2}} + {\text{2H}}_{{2}} {\text{O}} + {\text{O}}_{{2}} \to {\text{4Fe}}\left( {{\text{OH}}} \right)_{{3}} \downarrow $$
- Cathodic:
  5 $$ {\text{2H}}^{ + } + {\text{2e}}^{ - } \to {\text{H}}_{{2}} \uparrow $$

As shown in Eqs. (3) and (5), the OH^−^ and H^+^ were consumed, which caused the pH value decreased in the anodic region and increased in the cathodic region of the HWBM.

### Change in the surface of samples

Figure 5 shows the surface morphology at both ends of the samples after the electrochemical treatment. It can be observed that the iron oxide was attached to the anodic surface of the samples, while obvious cracks formed on the cathodic surface. This phenomenon is due to the oxidation reaction occurring in the anodic region of the samples and the reduction reaction occurring in the cathodic region. The reduction reaction caused the temperature of the cathode region to drop, Then, the combined action of the cold-contraction and release of hydrogen caused the cracks to form.

### UCS of the samples

**Figure 6:**
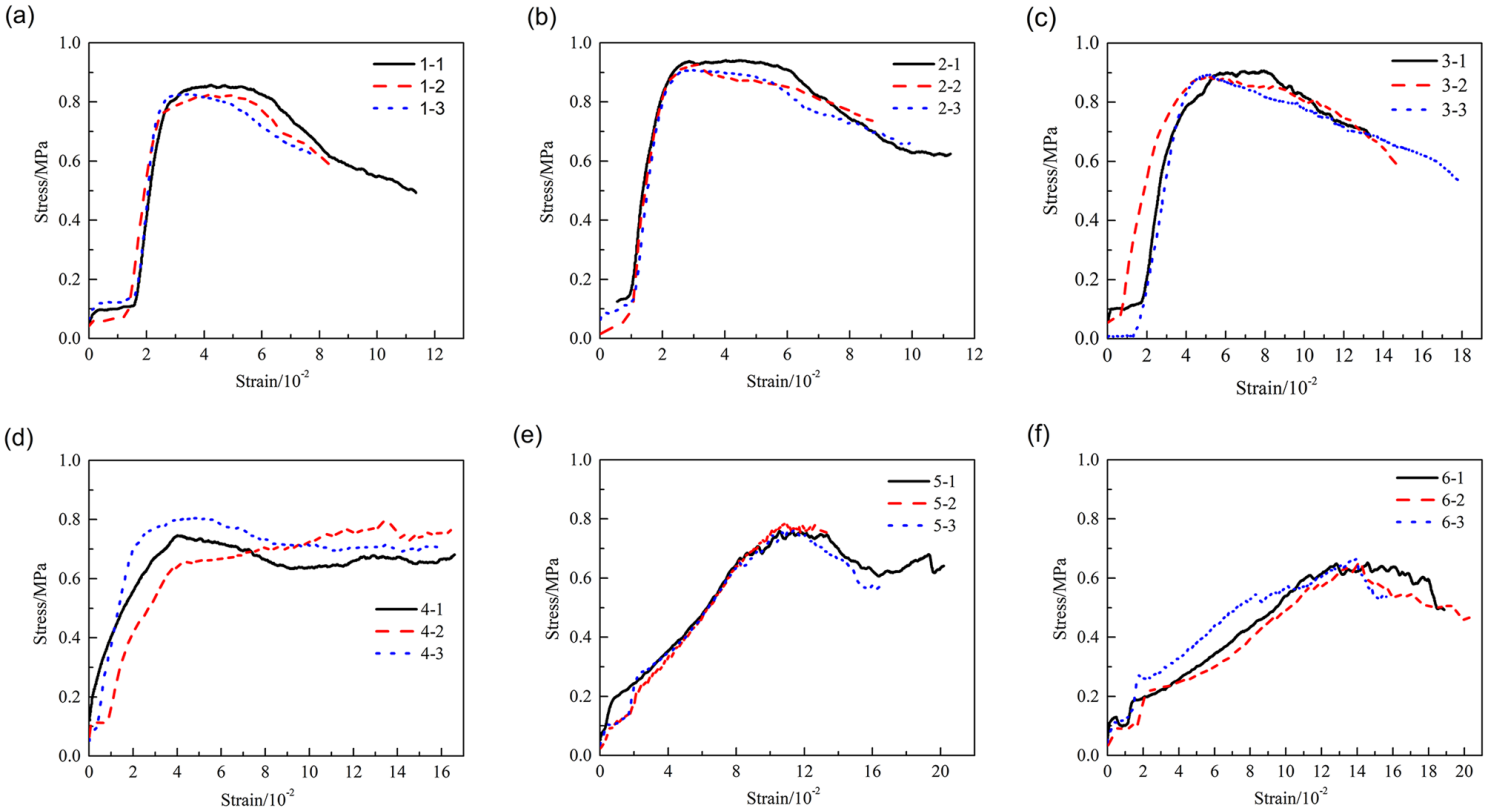
The stress–strain curves of samples before and after electrochemical treatment: (**a**) untreated samples; (**b**) 0.2 V/cm; (**c**) 0.5 V/cm; (**d**) 1.0 V/cm; (**e**) 2.0 V/cm; (f) 3.0 V/cm.

The uniaxial compressive stress–strain curves obtained for the HWBM samples after receiving the electrochemical treatment with different potential gradients are shown in Fig. 6.

The stress–strain curves shown in Fig. 6 indicate that the HWBM samples had a definite residual strength after the peak stress was reached, and the residual strength of the sample increased after the electrochemical treatment with an increasing potential gradient. Additionally, with an increasing potential gradient from 0.2 V/cm to 0.5 V/cm, 1.0 V/cm, 2.0 V/cm and 3.0 V/cm, the residual strain of the samples increased after the electrochemical treatment.

**Figure 7:**
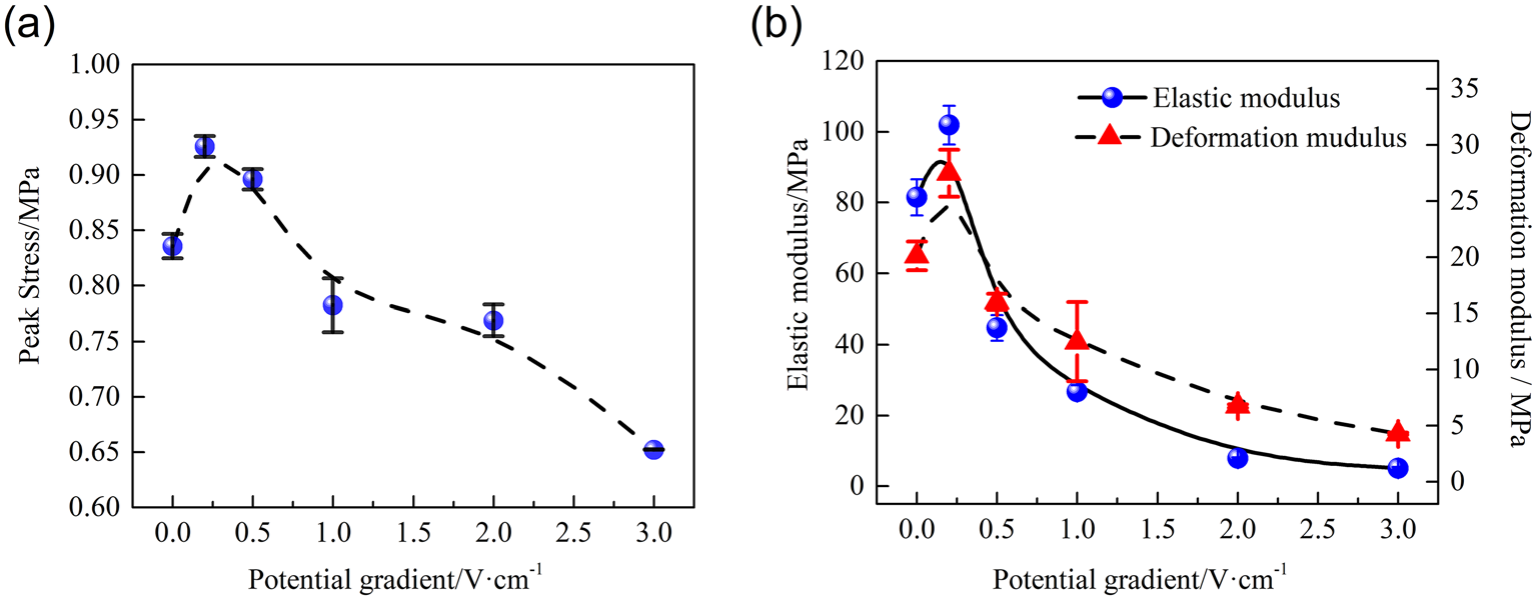
The mechanic properties of the HWBM samples after electrochemical treatment with different potential gradients. (**a**) Peak strength; (**b**) Elastic modulus and deformation modulus.

To further investigate the influence of the potential gradient on the electrochemical modification efficiency of the HWBM, the peak strength, deformation modulus and elastic modulus of the HWBM samples were calculated. Figure 7a,b show the influence of the different potential gradients on the peak strength, deformation modulus, and elastic modulus of the HWBM samples after the electrochemical treatment. Compared with the untreated samples, the mechanical properties of the samples after electrochemical treatment with different potential gradients changed to different degrees, and the following observations were made:

1. When the potential gradient was 0.2 V/cm and 0.5 V/cm, the UCS of the HWBM increased by 10.79% and 7.22% respectively; when the potential gradient was 1.0 V/cm, 2.0 V/cm and 3.0 V/cm, the UCS of the HWBM decreased by 6.37%, 7.99% and 21.98% respectively. This result indicates that treatment with a weak DC electric field (less than 0.5 V/cm) can reinforce the UCS of HWBMs.
2. When the potential gradient was 0.2 V/cm, the elastic modulus of the samples increased by 25.01%; when the potential gradient increased from 0.5 V/cm to 1.0 V/cm, 2.0 V/cm, and 3.0 V/cm, the elastic modulus of the samples decreased by 45.12%, 67.26%, 90.35%, and 93.72% respectively. This result indicates that the electrochemical treatment can alter the elastic modulus of HWBMs significantly, while the potential gradient of 0.2 V/cm can improve the elastic modulus of samples.
3. When the potential gradient was 0.2 V/cm, the deformation modulus of the samples increased by 36.78%; when the potential gradient increased from 0.5 V/cm to 1.0 V/cm, 2.0 V/cm, and 3.0 V/cm, the deformation modulus of the samples decreased from 20.29%, 37.96%, 66.38%, and 78.78% respectively. It indicates that the deformation modulus of the HWBM was modified by electrochemical treatment. The potential gradient of 0.2 V/cm can improve the deformation modulus of the HWBM efficiently.

In summary, according to the change in the peak strength, elastic modulus and deformation modulus of the samples after receiving electrochemical treatments with different potential gradients, 0.2 V/cm is the optimal potential gradient for electrochemically modifying the HWBM, which can simultaneously improve the compression strength and plastic deformation capacity of the HWBM.

### Electrical resistivity change of samples

To investigate the relationship between electrical resistivity and UCS of HWBMs, the volume electrical resistivity of the samples was calculated.

**Figure 8:**
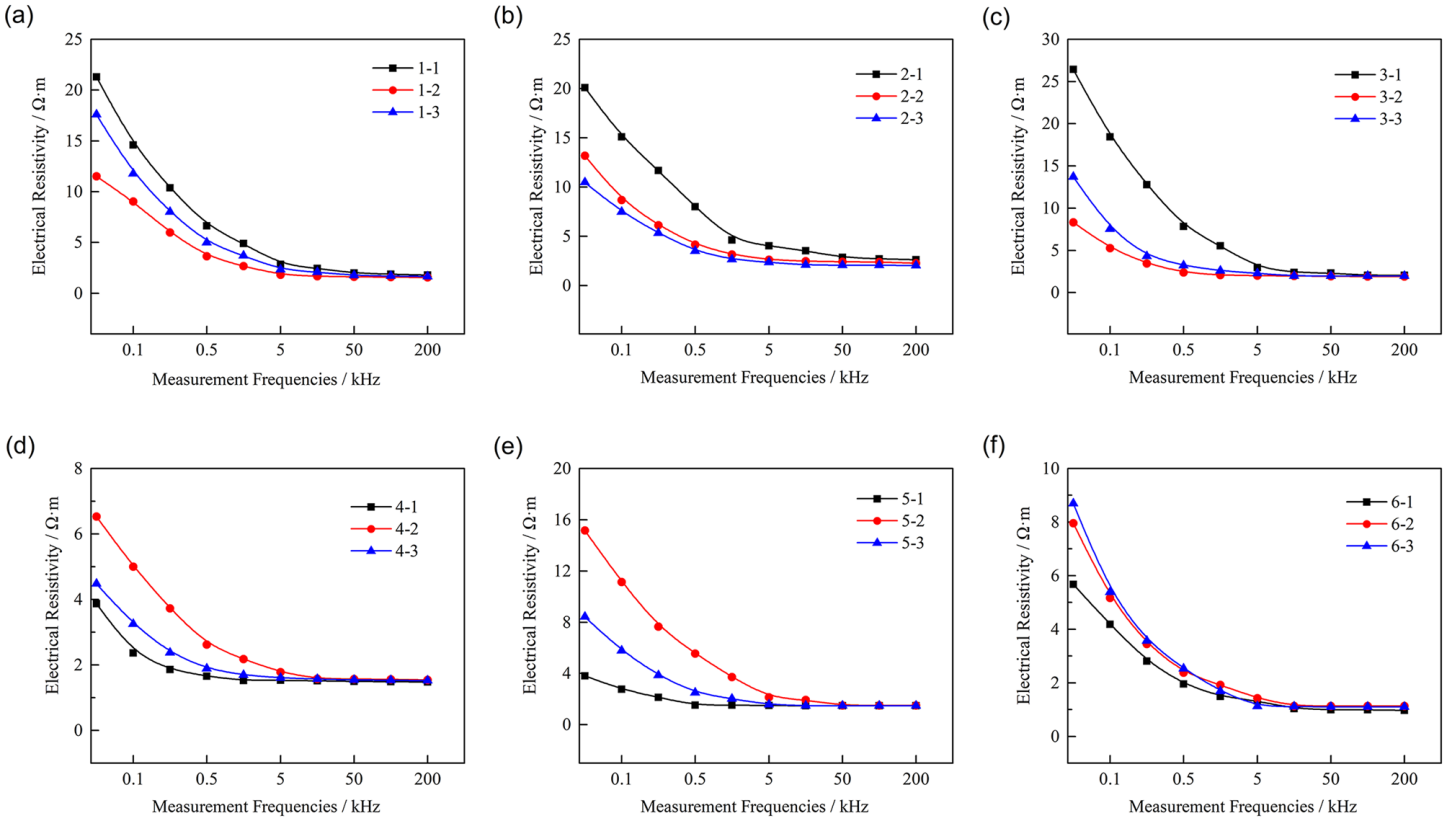
Electrical resistivity measurement curves of the HWBM samples: (**a**) untreated samples and samples receiving treatments with a potential gradient of (**b**) 0.2 V/cm, (**c**) 0.5 V/cm, (**d**) 1.0 V/cm, (**e**) 2.0 V/cm, (**f**) 3.0 V/cm.

Figure 8 shows the electrical resistivity measurement curves of the samples at different measurement frequencies. It can be observed that the electrical resistivityof the samples first decreased rapidly when the measurement frequency increased from 50 Hz to 5 kHz, then decreased slowly, and finally tended to be stable when the measurement frequency was increased from 5 to 200 kHz. This result is consistent with the research on zinc- contaminated soil solidified by cement^30^. The electrical resistivity measurements indicate that the frequency used to measure the electrical resistivity of HWBMs should exceed 5 kHz.

Therefore, in this work, the electrical resistivity of the HWBM samples was measured at 200 kHz and used to investigate the effects of electrochemical treatments with different potential gradients on the electrical resistivity of the resulting materials and the relationship between the UCS and electrical resistivity of HWBMs. The influence of the potential gradient on the average electrical resistivity of the samples after the electrochemical treatment was investigated.

**Figure 9:**
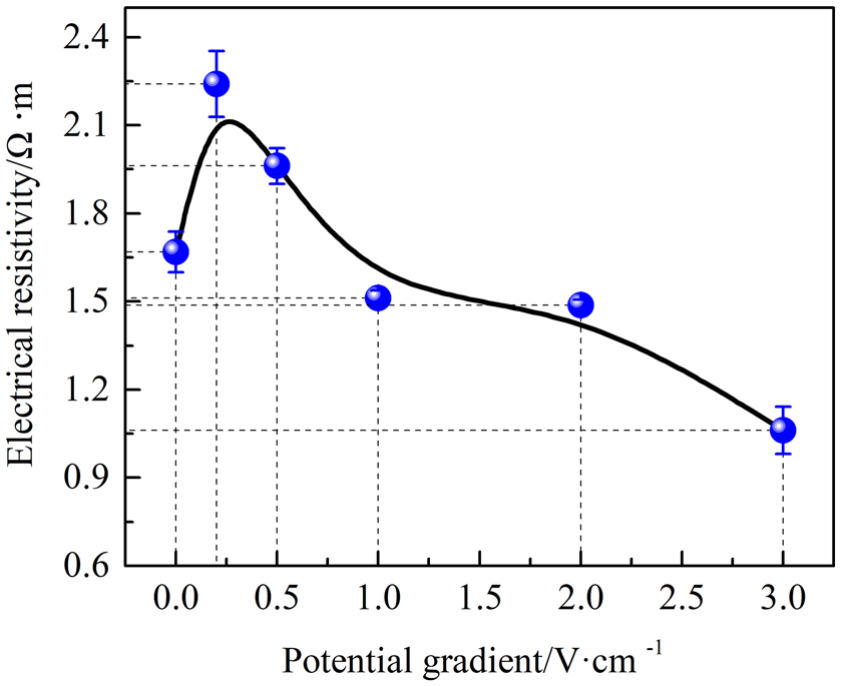
Change in the electrical resistivity after the electrochemical treatment was applied using a measurement frequency of 200 kHz.

As shown in Fig. 9, it can be seen that the electrical resistivity of the HWBM samples first increased and then decreased after the electrochemical treatment with the increasing of the potential gradient, and an inflection point was observed when the potential gradient was 0.2 V/cm. This result is basically consistent with the change in the uniaxial compression strength of the HWBM.

### Mineralogical composition change of the HWBMs

To investigate the mechanism responsible for the change in the strength and electrical resistivity of the HWBM receiving the electrochemical treatment, the mineralogical composition of the anodic region and cathodic region of the samples after receiving the electrochemical treatment with different potential gradients was measured as described in “XRD and SEM tests”.

**Figure 10:**
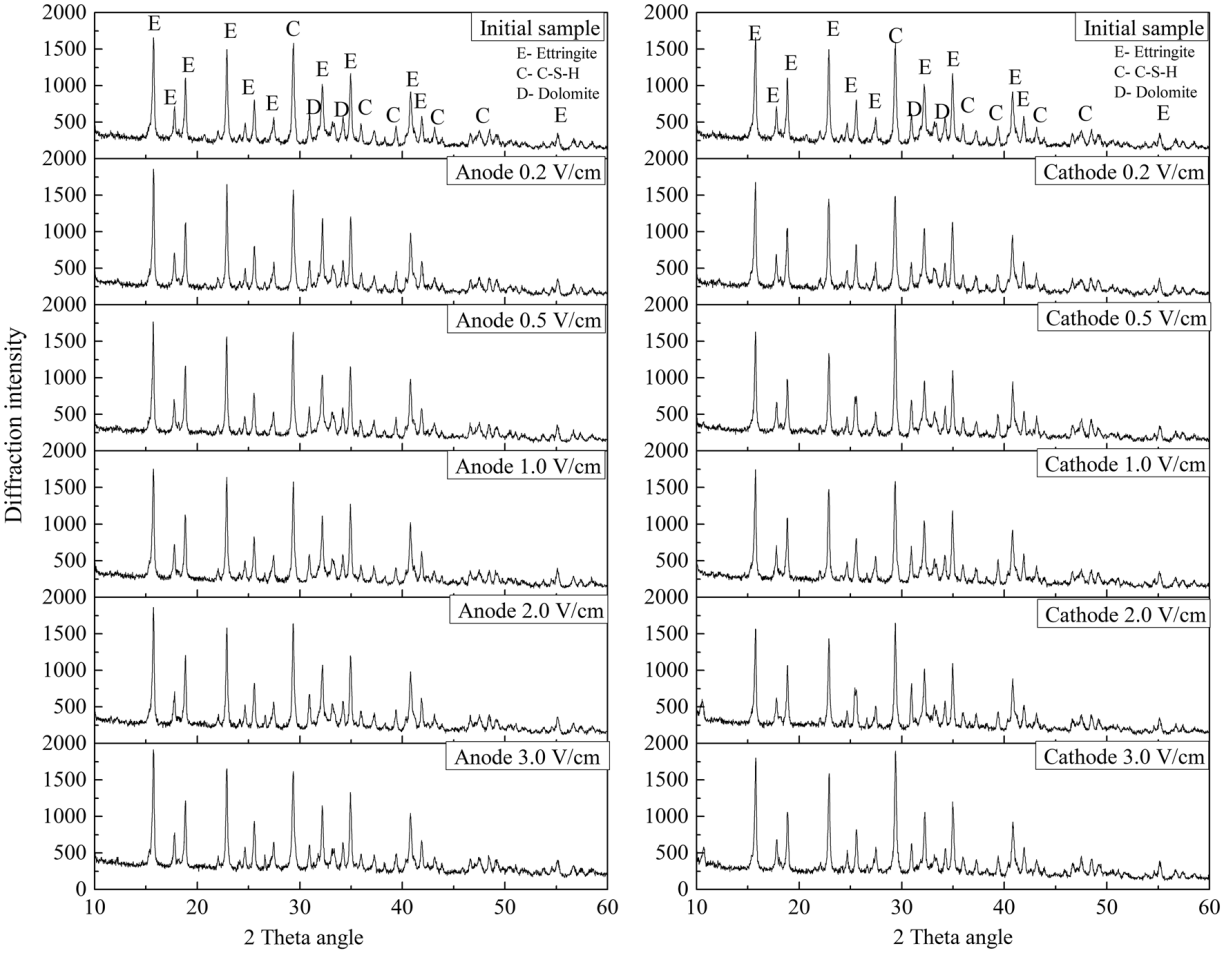
XRD patterns of HWBMs after electrochemical treatment with different potential gradients.

Figure 10 shows the XRD patterns of the anodic region and cathodic region of the HWBM samples after the electrochemical treatment with different potential gradients and after curing for 7 days. The main hydration product of the HWBM was ettringite, and there is some calcium silicate hydrates (C–S–H) generated as well. After the electrochemical treatment was applied with different potential gradients, the diffraction intensity of the peaks corresponding to ettringite and C–S–H did not obviously change in the anodic region, while the intensity of the diffraction peaks corresponding to ettringite and C–S–H did change significantly in the cathodic region. Meanwhile, the intensity of the diffraction peaks obtained for the anodic region was lower than those obtained for the cathodic region.

The abovementioned results indicate that the hydration of ettringite was restrained by the electrochemical treatment in the cathodic region of the samples, while the electrochemical treatment accelerated the formation of C–S–H in the cathodic region due to the increasing of the pH value. Thus, the semiquantitative analysis of the main phases was conducted to further explore the influence of the potential gradient on the electrochemical modification efficiency of the HWBM samples.

**Figure 11:**
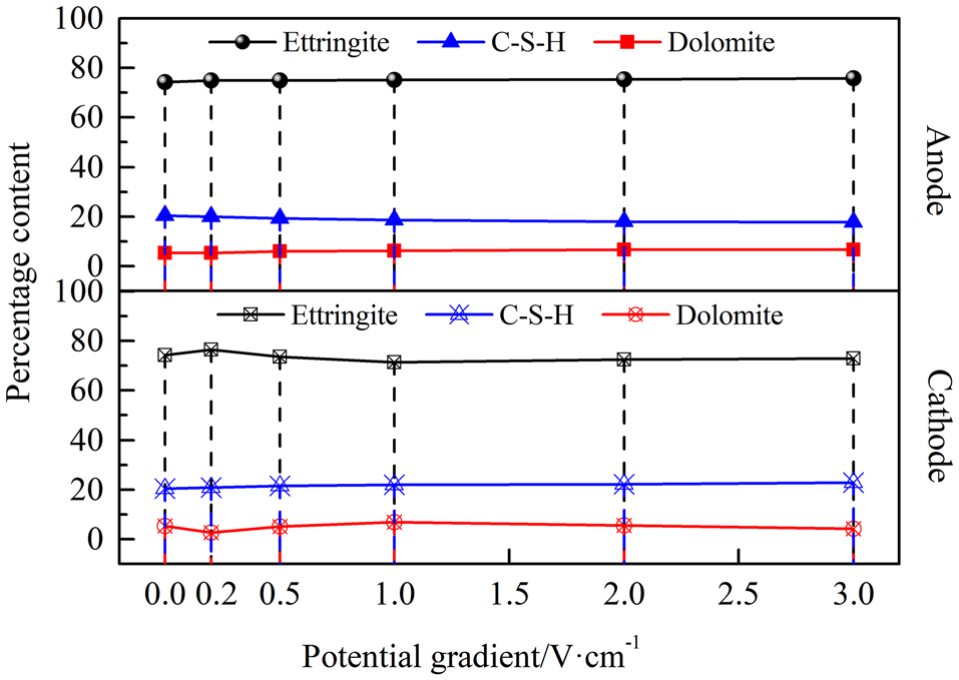
Change in the mineralogical composition of the samples after receiving electrochemical treatments with different potential gradients.

**Figure 12:**
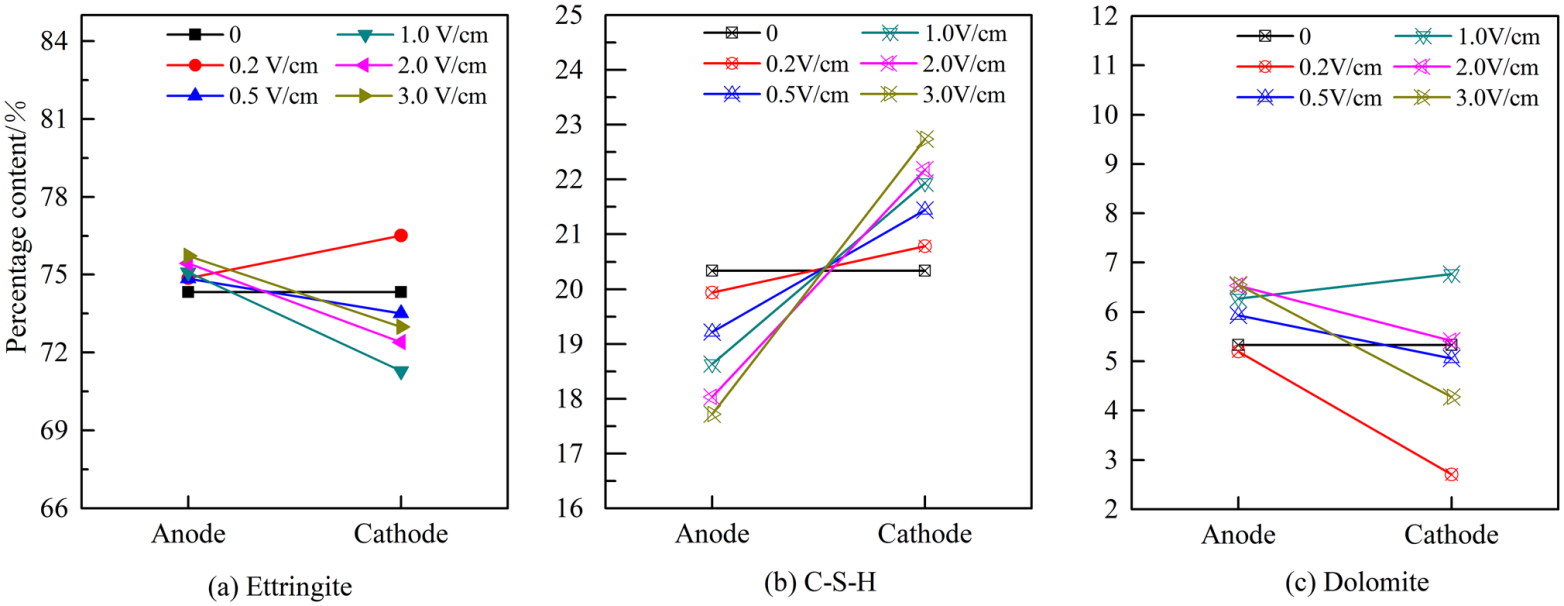
**C**hange in the different phases in the anodic and cathodic region of the HWBM samples after receiving electrochemical treatments with different potential gradients.

Figures 11 and 12 show that the effects of the different potential gradients on the hydration products of the anodic and cathodic regions of the HWBM samples after the electrochemical treatment.

The content and structure of ettringite and C–S–H is the main factor influencing the mechanic properties of the HWBM according to the previous reports^1,7,8^. These results indicate that there are different variation change on content of ettringite and C–S–H in the anodic region and cathodic region of samples after electrochemical treatment. However, the content of ettringite increased when the potential gradient was 0.2 V/cm, while the content of ettringite in cathodic region decreased significantly when exceeded 0.2 V/cm. Additionally, the content of C–S–H in the anodic region decreased, while increased in the cathodic region. Furthermore, with the increasing of the potential gradient, the anisotropy increased for the content of ettringite and C–S–H in the anodic and cathodic regions. Therefore, the abovementioned results prove that the change of content of ettringite and C–S–H in different regions caused the change in mechanic properties and electrical resistivity of the HWBM samples after the electrochemical treatment.

### Change in the microstructure of the HWBMs

**Figure 13:**
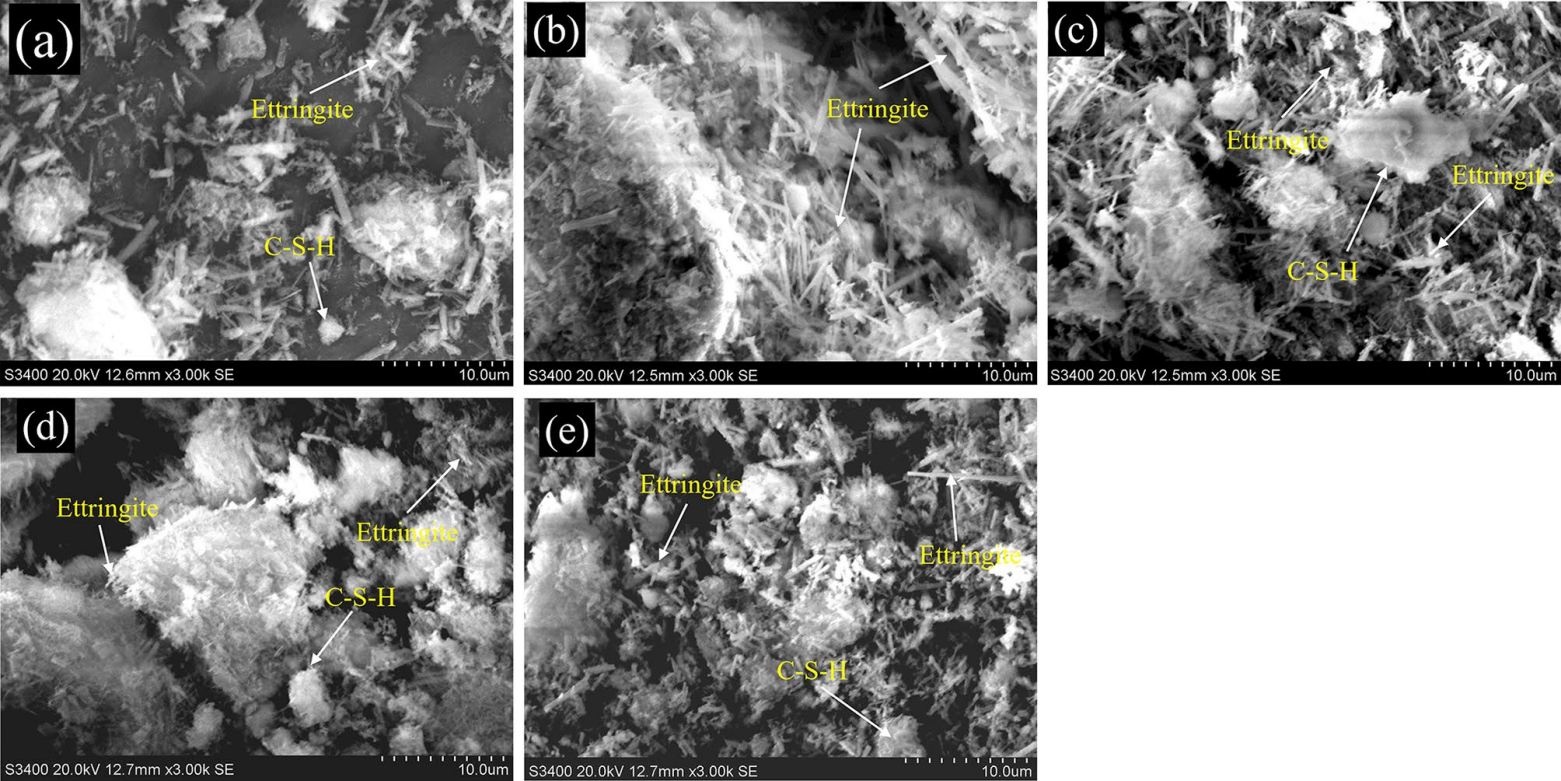
SEM images of the HWBMs after curing for 7 days: (**a**) untreated sample; (**b**) 0.2 V/cm anode; (**c**) 0.2 V/cm cathode; (**d**) 3.0 V/cm anode; (**e**) 3.0 V/cm cathode.

Figure 13 shows the SEM images of the anodic region and cathodic region of the samples after the electrochemical treatment. In this work, the images of the samples after electrochemical treatment when the potential gradient was 0.2 V/cm and 3.0 V/cm were selected to investigate the effect of the potential gradient on the hydration process and the microstructure of the hydration products. The SEM images show that the main hydration product of the HWBM is ettringite and C–S–H, while the shapes of ettringite are mainly acicular and columnar^1^. After the electrochemical treatment, the shapes and structures of ettringite obviously changed.

Figure 13a shows that the ettringite generated in the untreated samples after curing for 7 days is columnar, and its structure is slightly loose. In addition, amorphous C–S–H gel was generated in the samples, but the number of C–S–H is relatively small.

As shown in Fig. 13, the samples treated by the electrochemical process were compared to the untreated samples. (1) When the potential gradient was 0.2 V/cm, as shown in Fig. 13b,c, a stable framework formed in the anode region of the sample after the electrochemical treatment, while the structure of ettringite in the cathodic region was looser and there were obvious pores and fractures; (2) When the potential gradient was 3.0 V/cm, as shown in Fig. 13d,e, the microstructure of ettringite was looser, and the number of pores and fractures increased after the electrochemical treatment. Furthermore, the deterioration of the structure is more serious in the cathodic region.

Meanwhile, it was observed that a lower potential gradient accelerated the formation of stable structure of ettringite in the samples compared with a high potential gradient. However, the number of pores and cracks in the cathodic region significantly increased compared with in the anodic region after the electrochemical treatment.

Because the effect of the shape and amount of ettringite on the UCS, deformation modulus and elastic modulus of the HWBM is obvious^1,7^, the above mentioned results also prove the effect of the electrochemical treatment on the UCS, deformation modulus and elastic modulus of the samples.

## Discussion

As mentioned in “Mineralogical composition change of the HWBMs” and “Change in the microstructure of the HWBMs”, the main hydration processes in the HWBM are the formation of ettringite and C–S–H as in Eqs. (6–8)^1,8^.

6 $$ {\text{3CaO}} \cdot {\text{3Al}}_{{2}} {\text{O}}_{{3}} \cdot {\text{CaSO}}_{{4}} + {2}\left( {{\text{CaSO}}_{{4}} \cdot {\text{2H}}_{{2}} {\text{O}}} \right) + {\text{36H}}_{{2}} {\text{O}} \to {\text{3CaO}} \cdot {\text{Al}}_{{2}} {\text{O}}_{{3}} \cdot {\text{3CaSO}}_{{4}} \cdot {\text{32H}}_{{2}} {\text{O}} + {2}\left( {{\text{Al}}_{{2}} {\text{O}}_{{3}} \cdot {\text{3H}}_{{2}} {\text{O}}} \right) $$

7 $$ {\text{3CaO}} + {\text{Al}}_{{2}} {\text{O}}_{{3}} \cdot {\text{3H}}_{{2}} {\text{O}} + {3}\left( {{\text{CaSO}}_{{4}} \cdot {\text{2H}}_{{2}} {\text{O}}} \right) + {\text{23H}}_{{2}} {\text{O}} \to {\text{3CaO}} \cdot {\text{Al}}_{{2}} {\text{O}}_{{3}} \cdot {\text{3CaSO}}_{{4}} \cdot {\text{32H}}_{{2}} {\text{O}} $$

8 $$ {\text{2CaO}} \cdot {\text{SiO}}_{{2}} + {\text{nH}}_{{2}} {\text{O}} \to {\text{C}} - {\text{S}} - {\text{H}} + {\text{Ca}}\left( {{\text{OH}}} \right)_{{2}} $$

It indicates that the ion concentration and water content are the main factors to affect the hydration processes of the HWBM, which will further lead to the change in content and microstructure of ettringite and C–S–H. Finally, the mechanic properties of the HWBM will change. Meanwhile, the change of pH value also will affect the formation of ettringite and C–S–H^32^. Therefore, under the DC electric field, the migration of the internal charged particles (such as charge ion, elecrton and polar water molecules, etc.) of the HWBM will cause the variation of ion concentration, pH value and water content in differet regions, which will lead to the change of machanic properties of the HWBM.

In this work, the electrochemical treatment method was used to modify the mechanic properties of the HWBM. Under a direct electric field, the main reactions that occurred in the HWBM are hydration and electrokinetic processes. (1) On the hydration processes, the ion and free water content in the samples decreased with the formation of hydration products such as the ettringite and C–S–H, which causes the consolidation of the mixed slurry and the decline in the electric current; (2) Electroosmosis, electro migration and electrophoresis are the three different electrokinetic processes that occur in the samples^10–12,27^, and these electrokinetic processes cause the water cement ratio to increase in the anodic region and decrease in the cathodic region. This phenomenon is due to the migration of polar water molecules, cations and other positively charged particles from the anodic region to the cathodic region, which in turn causes the mechanic properties of the HWBMs. Additionally, it also caused the anisotropy of mechanic properties to increase between the anodic and cathodic region of the HWBM samples with the increasing of the potential gradient. As a result, the UCS, deformation modulus and elastic modulus of the samples after the electrochemical treatment first increase and then decrease with increasing the potential gradient.

Furthermore, electrolysis causes the formation of cracks in the cathode and corrosion in the anode, which may cause the UCS of the samples to decrease but the residual strength to increase due to the consolidation of iron oxide^33^. Meanwhile, electrolysis also causes the observed changes in the mineralogical composition of the HWBM. which further causes the mechanic properties and electrical resistivity of the HWBM to change because the content and structure of ettringite and C–S–H are the main factors influencing the mechanic properties of the HWBM. Generally, it is believed that the more ettringite and C–S–H there are, the higher UCS, deformation modulus and elastic modulus are^1,7,8^. The results of XRD and SEM in “Mineralogical composition change of the HWBMs” and “Change in the microstructure of the HWBMs” also indicate that the electrochemical treatment has obvious effect on the formation of the hydration products in the samples. However, with the increasing of the potential gradient, the anisotropy between the anodic and cathodic region of the samples increased, which caused the UCS, deformation modulus and elastic modulus of the HWBMs to first increase and then decrease after electrochemical modification. This result proves the effect of the electrochemical treatment on the UCS and electrical resistivity of the HWBM samples.

Therefore, the mechanic properties and electrical resistivity of the HWBMs changes as a resulting of applying an electrochemical treatment with different potential gradients, which is the result of the combined hydration and electrokinetic processes. In addition, considering the engineering demands, there is an optimal potential gradient for the electrochemical modification of HWBMs. In this work, when the potential gradient was 0.2 V/cm, the electrochemical method can significantly enhance the strength and deformability of the HWBM. However, the optimal potential gradient is different for different materials^31^. Thus, further experiments need to be performed to explore the electrochemical modification mechanism of the HWBMs and the influence of the electrochemical parameters (the potential gradient, electrode materials, electrolyte, pH, etc.) on the electrochemically modified effects. Additionally, the efficiency and cost of the electrochemical modification of HWBMs needs to be evaluated in the future.

Additionally, both the electrical resistivity and mechanical properties of rock and soil mass are inherently determined by the chemical composition, pore structure, and particle size, etc. of the rock and soil. Thus, there is possibly a strong relationship between the electrical resistivity and mechanical properties of rock and soil mass. A number of experiments, such as experiments to determine the linear correlation between the electrical resistivity and UCS of cemented soiland zinc-contaminated soil solidified by cement^34,35^, linear correlation between the electrical resistivity and the void ratio in undisturbed soil^36^, negative exponential relationship between the apparent resistivity and UCS in reinforced concrete^37^, and logarithmic relationship between the electrical resistivity and UCS in cemented backfill mass^38^, etc., have been carried out to demonstrate this correlation. However, there are no reports demonstrating the correlation between the electrical resistivity and UCS of HWBMs at present. Therefore, the relationship between the electrical resistivity and UCS of HWBMs was investigated in this paper to determine the UCS of HWBMs using the electrical resistivity.

**Figure 14:**
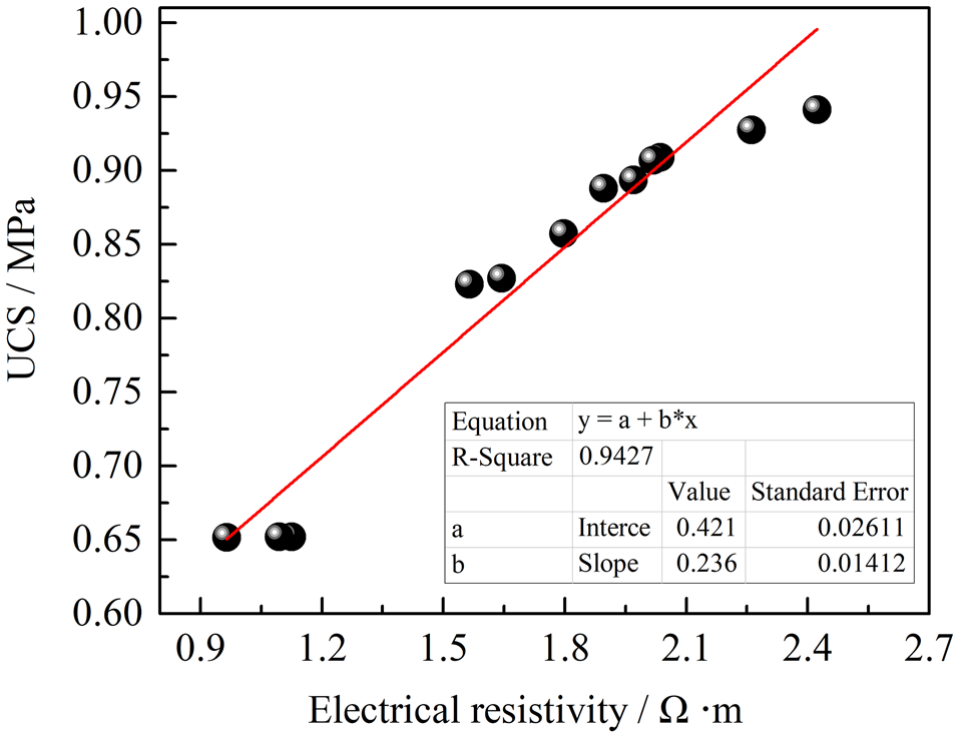
The relation curve of strength and electrical resistivity of HWBMs.

Figure 14 shows that there is a linear positive correlation between the averaged UCS and electrical resistivity of the HWBMs. However, this relationship is difficult to quantitatively characterize by a specific formula due to the limit of the small amount of test sample data. Thus, a large number of experiments need to be performed to explore the quantitative relationship between the UCS and the electrical resistivity of HWBMs, and the results can provide a theoretical and practical foundation for the nondestructive determination of rock and earth mass in the geotechnical engineering field.

## Conclusions

In this work, a series of experiments were carried out to investigate the influence of the electrochemical treatment on the mechanic properties, electrical resistivity, mineral composition and microstructure of HWBMs. The results are summarized as follows.

1. After the electrochemical treatment, the peak strength, elastic modulus and deformation modulus of the HWBM first increased and then decreased with increasing of the potential gradient, and the extreme point occurs when the potential gradient was 0.2 V/cm.
2. The electrical resistivity of the HWBM samples first increased and then decreased when an increasing potential gradient was applied during the electrochemical treatment, and the inflection point was observed to occur at 0.2 V/cm. Additionally, there is a positive correlation between the electrical resistivity and the UCS of the HWBMs.
3. The mineralogical composition of the samples was found to be unchanged. However, the content of ettringite and C–S–H was found to be significantly change in the anodic and cathodic region of the samples, and the anisotropy between anodic and cathodic region increased with the increasing of the potential gradient. Additionally, a more stable skeleton structure formed in the anodic region after the lower potential gradient electrochemical treatment, while more surface cracks formed in the cathodic region. However, when the potential gradient increases, the microstructure of the HWBM samples is loose and porous. This is the main factor influencing the change in the mechanical properties and electrical resistivity of the HWBM after the electrochemical treatment with different potential gradients.
